# Aberrant expression of calcitonin gene-related peptide and its correlation with prognosis in severe childhood pneumonia

**DOI:** 10.6061/clinics/2020/e1448

**Published:** 2020-01-20

**Authors:** Baoqin Tao, Lei Jiang, Liang Chen

**Affiliations:** I Emergency Department, Children's Hospital affiliated to Zhengzhou University, Zhengzhou, 450000 China; II Emergency Department, First Affiliated Hospital of Naval Medical University, Shanghai, 200433 China; III Department of Respiratory, Xiamen Chang Gung Hospital Xiamen, Fujian Province, 361028 China

**Keywords:** Calcitonin Gene-Related Peptide, Children, Severe Pneumonia

## Abstract

**OBJECTIVES:**

The purpose of this study was to evaluate the relationship between the serum levels of calcitonin gene-related peptide (CGRP) and the prognosis of pediatric patients with severe pneumonia.

**METHODS:**

Children diagnosed with severe pneumonia (n=76) were stratified into the survival (n=58) and non-survival groups (n=18) according to their 28-day survival status and into the non-risk (n=51), risk (n=17) and high-risk (n=8) categories based on the pediatric critical illness score (PCIS). Demographic data and laboratory results were collected. Serum CGRP levels were determined by enzyme-linked immunosorbent assay (ELISA). A receiver operating characteristic (ROC) curve was generated to determine the cutoff score for high CGRP levels.

**RESULTS:**

Serum CGRP levels were significantly higher in the survival group than in the non-survival group and were significantly higher in the non-risk group than in the risk and high-risk groups. The ROC curve for the prognostic potential of CGRP yielded a significant area under the curve (AUC) value with considerable sensitivity and specificity.

**CONCLUSION:**

Our findings show that CGRP downregulation might be a diagnostic marker that predicts the prognosis and survival of children with severe pneumonia.

## INTRODUCTION

Respiratory tract infections play an important role in pediatric diseases, result in serious illness, and have a high incidence rate (1). In recent years, with continuous improvements in living conditions, health care awareness, and medical and health conditions, the incidence of infectious diseases in infants and young children continues to decline. Respiratory infection is still the most common disease in children (2). Pneumonia remains the leading cause of death among children in China. Children are more prone to infectious respiratory diseases because of the anatomical and physiological characteristics of the respiratory system in early childhood. Respiratory infection causes mucus obstruction, leading to interstitial pneumonia, emphysema, atelectasis and so on (3). Pneumonia refers to inflammation in the final airway, alveoli and interstitium of the lungs caused by pathogenic microorganisms, physical and chemical factors, immune injury, allergy and antibiotics (4). It is a common infectious disease that seriously threatens human health. Patients with severe pneumonia may suffer from related clinical symptoms involving the circulatory, nervous and digestive tract systems. Early diagnosis and treatment can reduce mortality; therefore, an appropriate biomarker would be helpful for diagnosis and to guide treatment.

Calcitonin gene-related peptide (CGRP), a sensory neuropeptide composed of 37 amino acids, is a novel member of the calcitonin family of peptides that includes adrenomedullin, islet amyloid polypeptides (IAPPs), calcitonin, and calcitonin receptor-stimulating peptides (CRSPs) (5). CGRP, which is encoded by the same gene that encodes calcitonin, was first discovered in humans and other mammals by Amara et al. in 1982 (6). CGRP was recently shown to be widely distributed in the nervous system, cardiovascular system and lungs (7). In recent years, numerous studies have provided strong evidence that CGRP possesses many biological activities, including in neuronal regeneration, endothelium-dependent vasodilation, the inhibition of vascular smooth muscle proliferation, and bone growth (8,9). In addition, other studies have indicated that CGRP may be involved in the pathophysiology of pulmonary diseases. A large body of evidence indicates that CGRP protects alveolar epithelial type II cells against hyperoxia-induced oxidative stress injury, DNA damage and apoptosis (10,11). Moreover, Norton et al. reported that CGRP hyperpolarizes pulmonary artery endothelial cells and smooth muscle cells (SMCs) via the activation of ATP-sensitive K^+^ (KATP) channels (12). Augustyniak et al. discovered that CGRP significantly suppresses *M. catarrhalis*-triggered alveolar epithelial type II cell apoptosis and neutrophil degranulation (13). However, the expression levels of CGRP and its relationship with prognosis in severe childhood pneumonia have not previously been examined.

## MATERIALS AND METHODS

### Patients

A total of 76 sequential children aged <3 years, diagnosed with severe pneumonia and hospitalized at our department between December 2013 and June 2017 were included in this study. The inclusion criteria based on the diagnostic criteria for severe childhood pneumonia were as follows: chest wall depression and severe respiratory distress; subjective symptoms of central cyanosis, apastia, dehydration and diminished consciousness; and the progression of hypoxemia and intrapulmonary and extrapulmonary complications. Patients with malignant tumors, malnutrition, autoimmune diseases, or severe heart, liver or kidney failure were excluded. All patients were initially divided into a survival group (n=58) and a non-survival group (n=18) according to their 28-day survival status. Their pediatric critical illness score (PCIS) was determined by two attending physicians independently within 24 hours after admission, and the results were averaged. The total possible score is 100 points, and the severity of pneumonia is ranked as follows: >80 points, noncritical; 71-80 points, critical; and ≤70 points, extremely critical. We then classified the patients into three subgroups based on the PCIS: non-risk (PCIS>80, n=51), risk (70<PCIS≤80, n=17) and high risk (PCIS≤70, n=8). The present study was approved by the Ethics Committee of Xiamen Chang Gung Hospital.

### Laboratory tests and clinical data

Demographic information, such as age, sex and the PCIS, was obtained within 24 hours of admission. Routine blood tests, including white blood cell (WBC) counts, were performed using an automatic biochemical analyzer (Beckman Coulter, Inc., Brea, CA, USA).

### Measurement of serum CGRP

Peripheral venous blood was drawn after admission and collected in plastic tubes containing ethylenediaminetetraacetic acid (EDTA) as an anticoagulant plus aprotinin (500 KIU/mL blood; Trasylol, Bayer, Leverkusen, Germany). Serum samples were isolated by centrifugation at 3000 rpm for 10 minutes and stored at -20°C. Serum CGRP levels were measured by enzyme-linked immunosorbent assay (ELISA) using a commercial ELISA kit (R&D Systems, Minneapolis, MN, USA) according to the manufacturer’s protocol. The absorbance at 450 nm was measured using a microplate reader.

### Statistical analysis

All data are expressed as the mean ± standard deviation (SD). Statistical analysis was performed using SPSS version 22.0 (SPSS Inc., Chicago, IL, USA). Differences between groups were analyzed using Student’s *t*-test. Receiver operating characteristic (ROC) curves were constructed using XLSTAT software. *p*<0.05 was considered to indicate statistical significance.

## RESULTS

### CGRP was highly expressed in the survival group

**Figure 1:**
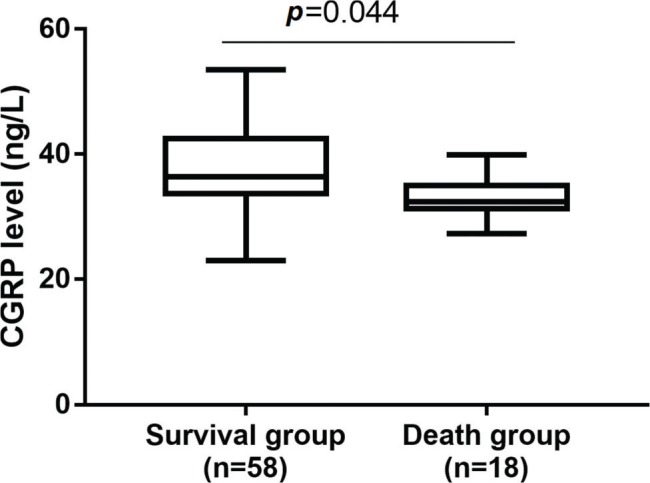
Serum CGRP levels in survival group (n=58) and non-survival group (n=18) determined by ELISA.

Seventy-six patients with severe pneumonia were hospitalized during the study period. These patients had a mean age of 18.3±9.8 months (range, 1 to 36 months) and included 42 (55.2%) males and 34 (44.7%) females. After 28 days, the survival rate was 76.31% (58 patients). A comparison of the clinical characteristics of the survival (n=58) and non-survival (n=18) groups revealed no differences in sex, age, PCIS or WBC count (Table 1, *p*>0.05). Compared with the non-survival group, the survival group had significantly increased serum levels of CGRP (Figure 1, *p*<0.05).

### CGRP was significantly increased in non-risk patients

**Figure 2:**
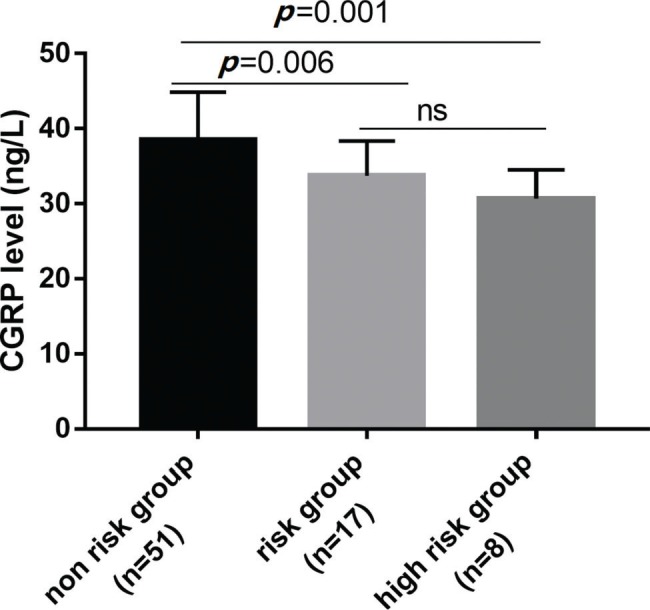
Serum CGRP levels in children with non risk, risk and high risk of severe pneumonia.

**Figure 3:**
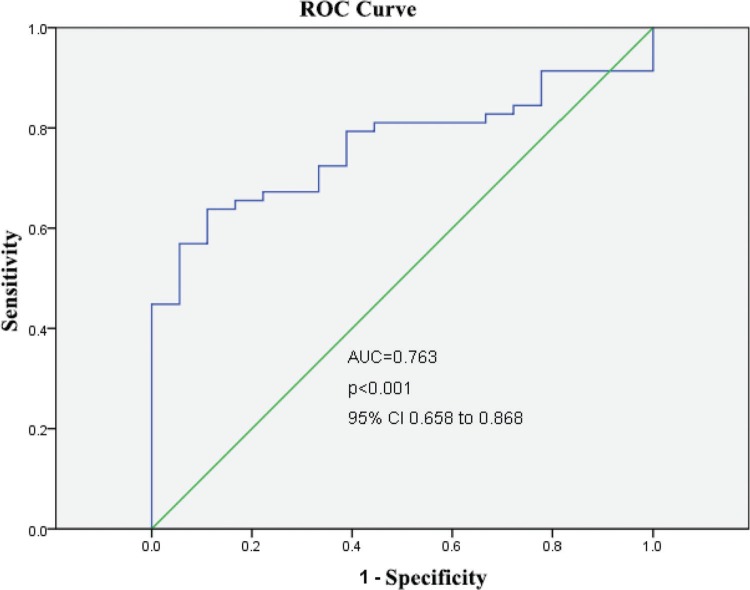
ROC curve for serum CGRP exhibiting the prognostic potential of CGRP in severe childhood pneumonia.

After categorizing the patients into the 3 risk groups according to the PCIS, there were 51 patients (females/males, 28/23) in the non-risk group, 17 (females/males, 9/8) in the risk group, and 8 (females/males, 5/3) in the high-risk group. The average age was 18.4 months in the non-risk group, 19.1 months in the risk group, and 15.9 months in the high-risk group (Table 2). The serum levels of CGRP in each risk group were further studied. Serum CGRP levels were markedly decreased in the risk and high-risk groups compared with the non-risk group (all *p*<0.05) and were similar in the risk and high-risk groups (*p*>0.05) (Figure 2). Furthermore, ROC curves were generated to verify the diagnostic role of serum CGRP, which yielded an area under the curve (AUC) of 0.763 (95%*CI*: 0.658-0.868), a sensitivity of 84.5%, and a specificity of 72.2% at the optimal cutoff value of 30 ng/L (Figure 3).

## DISCUSSION

Severe pneumonia, the leading cause of mortality in children under 5 years old, is characterized by hypoxemia, central cyanosis, severe respiratory distress, diminished consciousness, apastia and dehydration (14). Recently, a few prognostic biomarkers that appear after the onset of severe pneumonia have been reported. For example, Lee et al. conducted a retrospective study of 152 adult patients with severe pneumonia, including community-acquired pneumonia (CAP), health care-associated pneumonia (HCAP), and hospital-acquired pneumonia (HAP), and found a significant correlation between elevated cardiac troponin (cTn) levels and mortality (15). Yang et al. showed that decreased serum YKL-40 levels after 5 days of standard therapy were an independent risk factor for viral pneumonia in children diagnosed with CAP (16). At present, we commonly use the PCIS to evaluate the initial condition of critically ill children upon admission to the pediatric intensive care unit (PICU) (17); however, whether this score can determine the prognosis remains controversial. Therefore, it is important to identify meaningful prognostic indicators of severe pneumonia at the early stage.

Cardioprotective and neuroprotective actions of CGRP upregulation have been suggested. For instance, Guo et al. stated that CGRP effectively reversed the hypoxia/reoxygenation (H/R)-induced reduction in mitochondrial membrane potential and suppressed cytosolic reactive oxygen species (ROS) and myocardial apoptosis (18). Furthermore, Singh and colleagues reported that the exogenous administration of CGRP suppressed macrophage infiltration and inflammatory cytokine secretion, which attenuated Alzheimer's disease (AD), suggesting that CGRP is a potential candidate target for the treatment of AD (19). CGRP has also been implicated in the regulation of inflammation, proliferation and fibrosis in pulmonary diseases such as pulmonary arterial hypertension (PAH), asthma, acute lung injury (ALI) and pulmonary fibrosis (20-23). However, its prognostic value in severe pneumonia is poorly understood. Our study first showed that serum CGRP levels are associated with mortality in patients with severe pneumonia after adjusting for age, sex, PCIS and WBC count at admission, suggesting that CGRP may be a novel risk factor for mortality in children with severe pneumonia. According to our results, assessing serum CGRP levels in children with severe pneumonia provides additional prognostic information beyond conventional risk scoring. The majority of our patients were in the non-risk group (PCIS>80), whereas approximately 22.4% and 10.5% of the patients were classified into the risk and high-risk groups, respectively. The risk and high-risk groups showed decreased serum CGRP levels relative to the non-risk group. To determine the cutoff score for CGRP positivity, ROC analysis was conducted, and survival status was evaluated. Serum samples with a score above the determined cutoff value were was considered to have high CGRP levels.

In summary, the present study indicated that serum CGRP levels are potentially clinically useful as a prognostic indicator after the onset of severe childhood pneumonia. However, there are still several limitations of this study. First, we could not determine CGRP levels in bronchoalveolar lavage fluid (BALF) due to the difficulty in obtaining samples. Second, we could not compare serum CGRP levels in patients with severe pneumonia and in healthy controls. More prospective clinical trials are needed to verify our findings. Third, the association between serum CGRP levels and mortality was not analyzed using a multivariate model in this study. Further investigations in larger patient populations are required to establish whether reduced CGRP levels are an independent mortality risk factor for severe pneumonia.

Highlights

1. CGRP was significantly increased in the survival group than those in the non-survival group.
2. CGRP was highly expressed in non risk group compared with risk and high risk groups.
3. Serum CGRP yielded AUC with a considerable sensitivity and specificity.

## AUTHOR CONTRIBUTIONS

Tao B and Jiang L designed the study, conducted most of the experiments and wrote the manuscript. Tao B and Chen L conducted the experiments and analyzed the data. All authors have read and approved the final version of the manuscript.

## Figures and Tables

**Table 1:** Comparison of demographic data between the survival and non-survival groups (mean±SD).

Group	Cases	Females/Males	Age (months)	PCIS	WBC count (×10^9^/L)
Survival	58	32/26	19.1±10.3	80.49±6.53	12.14±2.36
Non-survival	18	10/8	15.9±7.8	81.50±7.60	11.97±1.63

**Table 2:** Comparison of sex and age among the non-risk, risk and high-risk groups.

Group	Cases	Females/Males	Age (months)
non-risk	51	28/23	18.4±9.5
risk	17	9/8	19.1±11.1
high-risk	8	5/3	15.9±9.4
